# Precarious work and its impact on health: a study of female textile workers in Lahore and Faisalabad

**DOI:** 10.3389/fpubh.2025.1529594

**Published:** 2025-04-14

**Authors:** Muhammad Khan, Rafi Amir-ud-Din, Rao Muhammad Atif

**Affiliations:** Department of Economics, COMSATS University Islamabad, Lahore, Pakistan

**Keywords:** textile industry, occupational diseases, wages and salaries, social security, occupational health, employment

## Abstract

**Introduction:**

This study aimed to examine work conditions, wage disparities, and health outcomes among female textile workers in Lahore and Faisalabad districts, employing a cross-sectional design to survey 541 participants.

**Methods:**

Utilizing the Respiratory Health Questionnaire, validated with a Cronbach alpha of 0.7762, alongside OLS and Logit regression models, the research investigated the interplay between employment conditions and health risks.

**Results:**

The analysis revealed significant wage discrepancies, with permanent employees earning PKR 1,896 more on average than their temporary counterparts (*p* < 0.01), and identified a strong correlation between employment type, excessive work hours, and health issues. Permanent contracts were associated with a 2.4-fold increase in the likelihood of excessive work hours (*p* < 0.01), while higher wages and dustier work environments were positively correlated with longer working hours. Conversely, social security benefits were linked to reduced excessive work hours (OR = 0.060956, *p* < 0.01). Furthermore, precarious work conditions, notably overtime and temporary contracts, significantly elevated the risk of respiratory problems and other health issues, with overtime work markedly increasing the likelihood of adverse health outcomes, including blood phlegm (OR = 11.285, *p* < 0.01) and fatigue (OR = 7.579, *p* < 0.01).

**Discussion:**

The findings underscore the critical need for policy interventions to improve work conditions, enforce minimum wage and work hour standards, and ensure the provision of social security benefits to mitigate health risks among this vulnerable worker group.

## 1 Introduction

The textile and garment industry has witnessed a surge in the employment of female workers across developing Asian countries (1). Despite periods of decline in female employment within the industry, attributed to legislative changes such as the Maternity Benefit and the Prohibition of Night Work Acts, and the mechanization of the industry (2), women have remained a constant presence. In some regions, the industry is predominantly female, with a significant portion of the workforce comprising women in unskilled or semi-skilled positions (3). The informal textile sector in India demonstrates a high percentage of female participation (4).

The textile sector has historically played a pivotal role in employing a large number of women, influenced by a multitude of factors that interplay to shape this trend. One of the primary reasons is the societal perception that certain tasks within the textile industry are more “suitable” for women, a notion deeply rooted in gender stereotypes. This perception, coupled with the exploitation and discrimination that women face in terms of wages and working conditions (2), has made the sector a significant, albeit challenging, source of employment for women. Another advantage may be that work in textile firms often involves manual and assembly line tasks that require precision and repetition–characteristics deemed well-suited to women (5).

Additionally, companies capitalize on women's general willingness to work extended hours and their perceived adaptability to substandard workplace conditions (6). It is also noteworthy that women, in many cases, refrain from engaging in collective bargaining or labor union activities. Their avoidance of seeking permanent contracts allows employers greater flexibility in their hiring decisions, enabling them to adapt to fluctuating demand conditions by rapidly adjusting their workforce (5, 7, 8). However, contrary to the stereotype that women are chosen for their supposed docility, many have demonstrated militancy and independence, often opting to work in cities of their choice and resisting family pressure (9). This challenges the narrative and highlights women's agency within the sector.

A rising concern in this context is the implication of such employment conditions on the health and wellbeing of these women. Precarious working conditions, characterized by insecure job contracts and suppressed wages, may heighten the risk of occupational hazards within textile mills. For instance, the transient nature of short-term contracts might compel women to tolerate suboptimal working conditions and reduced wages. This could lead to detrimental alterations in their work behavior, such as prolonged working hours beyond their capacity. The resulting exhaustion, coupled with an endemic lack of social security benefits, health insurance, and other essential job benefits, exacerbates the risks to their mental and physical health (10, 11).

Moreover, the literature points to the textile industry's environmental footprint, particularly its contribution to indoor air pollution. Epidemiological studies have consistently identified a link between cotton dust exposure in textile mills and the onset of respiratory diseases (7, 11–13). One of the most notable conditions linked to this exposure is byssinosis–a debilitating lung disease that has a marked prevalence in developing countries, including several in Asia (14–20).

Despite the extensive research conducted in recent years highlighting the association between precarious employment and health, gaps remain in our understanding. The specific mechanisms underpinning how unstable employment conditions adversely affect health remain largely underexplored (21, 22). Although some scholars have developed multifaceted models to investigate the association between employment precarity and health outcomes (23), there is a compelling need for studies that further explore the nexus between unstable employment and diverse workplace behaviors. In the textile sector, the dual challenges women face–not just job instability but also health and safety risks stemming from inadequate safety measures and harmful dust–warrant comprehensive examination. Analyzing these intertwined vulnerabilities can show important patterns that currently hinder the textile industry's efforts to foster a safer and more stable work environment for its workers. In light of these considerations, this study has a dual objective: to explore precarious employment and working conditions in textile mills, and to evaluate their implications for worker health and safety.

## 2 Materials and methods

### 2.1 Development of the study instruments

The primary data collection tool employed in this research was the Respiratory Health Questionnaire (24). The questionnaire encompasses diverse areas, capturing details pertaining to the workers' socio-demographics, wage, working conditions, occupational exposures, employment type, contract duration, provisions for health insurance, and social security, among others. In terms of reliability, the instrument's validation statistics revealed a Cronbach α test score of 0.7762, confirming its consistency and reliability. A preliminary test of this questionnaire was conducted on a cohort of twenty textile spinning mill workers. Feedback from this pre-test led to minor modifications in the questionnaire to enhance its efficacy for the final data collection.

### 2.2 Sample size and sampling technique

This research employed a cross-sectional design and was executed across 18 textile mills located in the Faisalabad and Lahore districts of Pakistan. The study's sample consisted of 541 female workers. A systematic multistage random sampling technique was adopted to determine the participants. In the first stage, comprehensive data on the spinning mills situated in the Faisalabad and Lahore districts were procured from the All-Pakistan Textile Mills Association (APTMA). The Faisalabad district housed 46 mills, while Lahore accounted for 31. In the second stage, to ensure unbiased selection, a randomization technique was employed. Consequently, 11 spinning mills from Faisalabad and 7 from Lahore were randomly chosen. For the third and final stage, depending on the workforce size of the selected mills, between 15 to 25 workers were chosen from each, once again using a random selection method.

### 2.3 Recruitment of participants and consent procedure

The data collection phase transpired between April and June 2015. Each potential participant was thoroughly briefed about the study's objectives, the nature of the information sought, and the assurance of confidentiality. Only after obtaining written informed consent from the workers was the data collected. It's pertinent to mention that workers who refrained from providing written consent were excluded from the study.

### 2.4 Analytical models

#### 2.4.1 Work conditions and workers' wages

In this specification, the dependent variable is represented by the worker's monthly wage in Pakistan Rupee (PKR). Key independent variables include the type of employment contract, social security provisions, and dust levels at the workplace. Employment contract is a dichotomous variable: 1 signifies permanent/full-time employment, and 0 indicates insecure contracts like short-term agreements or daily wages. Likewise, social security is binary: 1 denotes its presence, 0 its absence. The proposed model for this analysis is an Ordinary Least Squares (OLS) regression, represented by the following equation:


(1) $$\begin{array}{l} \text{W}=\beta_{0}+\beta_{1}\text{EC}+\beta_{2}\text{DL}+\beta_{3}\text{SS}+\beta_{4}\text{A}+\beta_{5}\text{MS}+\beta_{6}\text{Edu}+\beta_{7}\text{TF}+\epsilon_{1} \end{array}$$

In Equation 1, *W* represents Wages, *EC* stands for Employment Contract, *DL* denotes Dust Level, *SS* is for Social Security, *A* signifies Age, *MS* represents Marital Status, *Edu* denotes Education, and *TF* stands for Type of Firm.

#### 2.4.2 Work conditions and excessive work hours

Here, the dependent variable, “excessive work hours,” is binary, where “1” indicates a weekly working duration exceeding 48 h, and “0” otherwise. Given the binary nature of the dependent variable, a Logit regression model is employed:


(2) $$\begin{array}{l} \text{EWH}=\alpha_{0}+\alpha_{1}\text{W}+\alpha_{2}\text{EC}+\alpha_{3}\text{DL}+\alpha_{4}\text{SS}+\alpha_{5}\text{A}+\alpha_{6}\text{MS}+\alpha_{7}\text{TF}+\epsilon_{2} \end{array}$$

In the Equation 2, *EWH* corresponds to Excessive Work Hours, with other terms such as *W* for Wages, *EC* for Employment Contract, and so on, consistent with the previous equation's abbreviations.

#### 2.4.3 Work conditions and occupational illness

For this analysis, the binary dependent variable is designed to estimate if a worker reports any form of physical or mental illness. The Logit regression model for Equation 3 is:


(3) $$\begin{array}{l} \text{I}=\gamma_{0}+\gamma_{1}\text{W}+\gamma_{2}\text{EC}+\gamma_{3}\text{EWH}+\gamma_{4}\text{OW}+\gamma_{5}\text{SS}+\gamma_{6}\text{DL}+\gamma_{7}\text{A}+\\ \;\gamma_{8}\text{MS}+\gamma_{9}\text{TF}+\gamma_{10}\text{SW}+\gamma_{11}\text{SK}+\epsilon_{3} \end{array}$$

For Equation 3, *I* stands for Illness, *EWH* denotes Excessive Work Hour, *OW* is for Overtime Work, *SW* represents Separate Washroom, *SK* signifies Separate Kitchen, and the other terms carry the meanings provided in the earlier sentences.

While textile industries involve similar dust types (e.g., cotton), exposure levels vary significantly across firms due to differences in ventilation, machinery, and safety practices (25). Our measurement of dust levels combined workplace observations and worker self-reports, categorized as “dusty” or “less dusty” (26). Though subjective, this approach aligns with the hedonic wage model (27), which posits that workers demand wage premiums for hazardous conditions. In Equation 1, dust level (*DL*) captures compensatory wage differentials, while in Equation 2, it reflects how hazardous environments may correlate with prolonged work hours due to productivity demands. Despite potential individual response biases, aggregate trends remain informative for policy targeting.

In Equation 3, the inclusion of a separate kitchen (SK) serves as a practical proxy for indoor air pollution, a known risk factor for respiratory illness (28). While fuel type or direct pollution metrics would be ideal, data limitations necessitated a simplified measure. Households without separate kitchens often experience concentrated cooking emissions, exacerbating respiratory symptoms. Although unmeasured confounders like secondhand smoke exist, our model controls for workplace dust (*DL*) and overtime (*OW*), isolating domestic contributions to illness. Future studies should integrate detailed air quality metrics, as noted in our limitations.

## 3 Results

Table 1 presents demographic and work-related data for women workers in the textile industry (*N* = 541). This table illustrates that the mean age of these workers is 26.23 years (SD = 7.11). Furthermore, an average family comprises 6.05 members, with a monthly household expenditure of PKR 19,196. Importantly, literacy rates among these workers are low, with 56% of them being illiterate. The data also provide insights into their employment conditions, indicating that the average wage is approximately PKR 10,816.45, with a standard deviation of 2,277.8. Surprisingly, the mean working duration exceeds the standard 48-hour workweek by 6 h, amounting to 54 h per week. A minority of these workers (9.2%) are on permanent employment contracts with fixed wages, and a significant majority (63%) do not have access to social security and labor benefits.

**Table 1:** Demographic and work characteristics of women workers (*N* = 541).

**Variables**	**Definition**	***N* (%)**	**Mean (SD)**
Wage per month	Pakistan Rupees (PKR)		10816.45 (2277.8)
Permanent employee	Yes	49 (9.2)	
Social security entitlement	Yes (if Social Security benefits are available)	195 (36.4)	
The workplace is more dusty	Yes (if the firm is more dusty compared to other similar firms)	16 (7.8)	
Work experience	Number of months in the textile job		44.25 (37.39)
Weekly work hours	Average work hours per week		53.92 (6.65)
Work overtime	Yes	64 (11)	
Firm type	Yes (for an exporting firm)	216 (39)	
Age	Years		26.23 (7.11)
Education	Years		2.38 (3.01)
Marital status	Married	74 (35.9	
Separate washroom	Yes (if there is a Separate washroom in the house)	487 (90)	
Separate kitchen	Yes (if there is a Separate kitchen in the house)	395 (73)	
Headache	Yes	119 (22)	
Dizziness	Yes	55 (10.9)	
Shortness of breath	Yes	86 (16)	
Fatigue	Yes	65 (12)	
Cough	Yes	114 (21)	
Cough with phlegm	Yes	58 (10.8)	
Cough with bloody phlegm	Yes	16 (3)	
Blood Pressure	Yes (if medically diagnosed)	81 (15)	

### 3.1 Work conditions, wages, and excessive hours

The regression results, as detailed in Table 2, focus on the determinants of wages and excessive work hours in textile mills. There is a significant association between the type of employment contract and the wages earned by these workers. On average, a permanently employed woman earns PKR 1896 (*p* < 0.01) more than her non-permanent counterpart. Factors such as work experience and age strongly influence wages. Additionally, single women tend to have higher earnings than married ones (*p* < 0.05).

**Table 2:** The determinants of wages and excessive work hours in textile mills.

**Dependent variable**	**Wages**	**Excessive work hours**
	β	**Odds ratio**
Employment contract (permanent)	1,896.85^***^	2.39971^**^
	(389.528)	(0.90989)
Wages		1.00032^***^
		(0.00054)
More Dusty (1 = yes, no = 0)	5.6970	1.42197^**^
	(147.088)	(0.25818)
Social security entitlement	110.65	-0.060956^***^
	(233.151)	(0.015708)
Type of firm	125.415	-3.947
	(214.606)	(1.3651)
Work Experience	15.477^***^	0.99849
	(2.700)	(0.00334)
Marital status (1 = married, 0 = others)	-267.118^**^	1.176
	(123.926)	(0.17637)
Education	59.483^*^	-0.98380
	(30.549)	(0.03558)
Age (years)	29.946^**^	-0.96893^*^
	(14.384)	(0.01650)
Constant	9410.304^***^	0.1776738^**^
	(537.746)	(0.139135)
Observations	541	541
R squared	0.1639	
Adjusted R squared	0.1514	
Pseudo R2		0.2554
LR chi2(8)		180.94
Prob > χ^2^		0.0000

Standard errors are in parenthesis.

* p < 0.10,

** p < 0.05,

*** p < 0.01.

The second model examines the factors associated with excessive work hours. Employment on a permanent contract increases the odds of excessive work hours by a factor of 2.39971 (*p* < 0.01). Higher wages are also positively associated with excessive work hours (odds ratio=1.00032, *p* < 0.01). Working in dustier environments corresponds with 1.42197 times higher odds of excessive work hours (*p* < 0.05). In contrast, having social security entitlement reduces the odds of excessive work hours by a factor of 0.060956 (*p* < 0.01). Other significant predictors include work experience, marital status, education, and age.

### 3.2 Work conditions and occupational illness

Table 3 examines the impact of precarious working conditions on various respiratory and health issues among textile mill workers. Employment on a temporary contract, as opposed to a permanent one, is associated with higher risks of shortness of breath (OR = 1.8139) and phlegm (OR = 1.4496), suggesting that job insecurity may exacerbate respiratory problems, though this effect is not statistically significant.

**Table 3:** The determinants of respiratory illness in textile mills.

**Dependent variables**	**SB**	**Ph**	**BlPh**	**DC**	**BP**	**HA**	**DZ**	**FT**
	**Odds ratios**							
Employment contract (permanent)	1.8139	1.4496	0.965418	0.5285	-0.39051	1.43800	-0.89742	0.59745
	(0.9284)	(0.7499)	(0.86967)	(0.2361)	(0.2327)	(0.6343)	(0.5650)	(0.3635)
Work overtime (1 = yes, no = 0)	2.0217^*^	2.380^*^	11.285^***^	1.264^*^	-.38087	3.729^***^	5.114^***^	7.579^***^
	(0.8388)	(0.985)	(8.295)	(0.402)	(0.4601)	(1.276)	(2.347)	(3.220)
Excessive hours (1 = yes, no = 0)	0.023043	-0.0012	1.0016	0.0147	0.03763	1.4187	2.24987^**^	1.2831
	(0.0104)	(0.011)	(0.019)	(0.010)	(0.020)	(0.305)	(0.8850)	(0.4718)
Wage	1.0002^***^	-0.1887	-0.03261	0.2209	-0.0220	0.99835	0.99997	1.0000
	(0.0006)	(0.095)	(0.048)	(0.163)	(0.088)	(0.0007)	(0.0000)	(0.0000)
Social security (1 = yes, no = 0)	0.1129	2.296^***^	-4.5910^***^	-0.675^***^	0.05663	0.92003	1.314535	0.64423
	(0.048)	(0.910)	(3.847)	(0.148)	(0.073)	(0.2782)	(0.5614)	(0.2634)
More Dusty (1 = yes, no = 0)	-0.1153	0.0055	-4.679	0.9323	0.0074^***^	0.4277	0.614^***^	0.84813
	(0.108)	(0.121)	(0.192)	(0.165)	(0.167)	(0.1305)	(0.1535)	(0.1986)
Work experience	0.993^***^	0.0004	1.0176^***^	0.2144	1.009^***^	1.0006	0.99438	0.99967
	(0.0037)	(0.191)	(0.0068)	(0.133)	(0.003)	(0.0032)	(0.0051)	(0.0045)
Cooking fuel (1=natural gas)	-0.1174	0.2758	-0.6220	0.0310	0.3484	0.3484	1.249	2.1607
	(0.184)	(0.210)	(0.495)	(0.1880)	(0.289)	(0.289)	(0.3823)	(0.669)
Separate kitchen (1 = yes, no = 0)	-0.4305	-0.514^***^	-0.1366	2.851^***^	0.4187	-0.492^***^	-0.36^***^	-0.78671
	(0.154)	(0.219)	(0.361)	(1.27)	(0.305)	(0.192)	(0.1694)	(0.4357)
Separate washroom (1 = yes, no = 0)	0.43725^*^	0.83865	4.06710	6.2051^*^	.219^***^	0.05663	3.168^*^	-0.289^***^
	(0.2027)	(0.617)	(6.7311)	(6.73)	(0.109)	(0.073)	(2.167)	(0.1618)
Marital status (1 = married, 0 = others)	0.0289	1.58^***^	1.1513	-0.6714^*^	-0.0297	1.10005	1.0335	1.459^***^
	(0.0886)	(0.297)	(0.148)	(0.1415)	(0.156)	(0.1725)	(0.1960)	(0.258)
Age (years)	1.046^***^	0.0152	1.0331	-0.0081	-0.0067	1.005727	1.0041	0.957^***^
	(0.0199)	(0.010)	(0.016)	(0.009)	(0.016)	(0.0172)	(0.0229)	(0.0243)
Constant	-0.02968^*^	-2.45^***^	0.00355^***^	0.02481^***^	0.109^***^	-0.31502	0.11349^*^	0.1671^*^
	(0.729)	(0.792)	(1.309)	(0.0333)	(0.1165)	(0.2824)	(0.1464)	(0.1944)
Observations	541	541	541	541	541	541	541	541
LR chi2(11)	38.80^***^	40.39^***^	40.80^***^	51.98^***^	8.98	33.07^***^	35.91^***^	53.79^***^
Log-likelihood	-225.690	-171.70	-58.507	-259.89	-77.805	-274.69	-168.441	-181.411
Pseudo R2	0.0792	0.108	0.2584	0.091	0.054	0.060	0.098	0.125

Standard errors are in parenthesis.

* p < 0.10,

** p < 0.05,

*** p < 0.01.

SB, Shortness of breath; Ph, Phlegm; BlPh, Blood phlegm; DC, Dry Cough; BP, Blood pressure; HA, Headache; DZ, Dizziness; FT, Fatigue.

Working overtime significantly increases the likelihood of multiple adverse health outcomes, including shortness of breath (OR = 2.0217, *p* < 0.1), phlegm (OR = 2.38, *p* < 0.1), blood phlegm (OR = 11.285, *p* < 0.01), dry cough (OR = 1.264, *p* < 0.1), headache (OR = 3.729, *p* < 0.01), dizziness (OR = 5.114, *p* < 0.01), and fatigue (OR = 7.579, *p* < 0.01). Excessive work hours are also linked to higher odds of blood phlegm (OR = 1.0016) and dizziness (OR = 2.24987, *p* < 0.05), highlighting the detrimental effects of long working hours on worker health.

Lower wages correspond with increased shortness of breath (OR = 1.0002, *p* < 0.01), indicating that economic precarity may exacerbate respiratory issues. Lack of social security further compounds health risks, with higher odds of blood phlegm (OR = 0.4591, *p* < 0.01) and dry cough (OR = 0.325, *p* < 0.01) for those without coverage.

Dustier working environments, a common hazard in textile mills, are associated with greater risks of blood pressure problems (OR = 1.0074, *p* < 0.01) but lower likelihood of dizziness (OR = 0.614, *p* < 0.01) and fatigue (OR = 0.84813). Longer work experience mitigates some risks like shortness of breath (OR = 0.993, *p* < 0.01) but increases others like blood phlegm (OR = 1.0176, *p* < 0.01) and blood pressure issues (OR = 1.009, *p* < 0.01).

The results underscore how precarious employment conditions, including temporary contracts, overtime, low wages, and lack of social protections, contribute to a range of adverse respiratory and general health outcomes among textile workers. Interventions addressing these workplace factors may be crucial for safeguarding worker wellbeing in this industry.

## 4 Discussion

This study analyzed the work organization within textile mills, particularly focusing on female workers, their wages, working conditions, and the consequent health implications. The data reveals that a significant proportion, 48% of respondents, receive wages that fall below the minimum wage standard of PKRs 12,000 for the year 2014–2015 (29). This underpayment is observed regardless of factors such as work experience or educational background, with affected workers earning approximately 30% less than the stipulated minimum wage.

The demographic data underscores the vulnerability of these workers: a majority are illiterate, hail from low-income families, and have limited alternative employment avenues outside of agriculture, where employment is often seasonal or without remuneration. The textile sector, therefore, emerges as a significant employer of this demographic. The abundant availability–or “elastic supply”–of such female laborers allows textile sector to manipulate labor market dynamics, often to the detriment of these workers by offering wages that may be discriminatory (29).

The study further highlights a disturbing aspect of their employment–extended working hours. The average working duration for these women stands at 54 h weekly, a clear deviation from the country's 48-hour workweek norm (30). This discrepancy could be attributed to the employers' strategy to circumvent minimum wage regulations by extending working hours. Given the limited bargaining power of these female workers, they often acquiesce to such conditions. Moreover, despite the extended work hours, an alarming 11% still engage in overtime to supplement their incomes.

However, the repercussions of such extended work hours are not merely economic. Prolonged durations of work in textile mills have been consistently linked with detrimental health outcomes (11, 13, 31). Studies demonstrate a significant correlation between the duration of employment in the textile industry and the prevalence of respiratory symptoms, notably in roles related to the spinning section (18). Beyond physical health, extended work hours exacerbate work-related rumination, negatively affecting employees' ability to recover during non-work hours. This persistent cognitive engagement with work tasks has been directly linked to diminished health outcomes (32).

Excessive work hours have also critical health implications on cardiovascular health. The relationship between long working hours and the risk of stroke, particularly haemorrhagic stroke, remains significant even after adjusting for cardiovascular risk factors (33). Prolonged work schedules have also been implicated in worsening mental health outcomes, as evidenced by higher instances of depressive symptoms among medical staff. The mediation effect of job burnout and the moderating role of social support reveal complex interactions between work hours, occupational stress, and mental health (34). The impact of long working hours on mental health has been observed globally, with studies from China indicating a significant association between extended work durations and the risk of mental illness, highlighting the vulnerability of women, white-collar workers, and employees in micro-firms (35).

In the current study, temporary contractual employment was associated with elevated risks of respiratory symptoms–specifically shortness of breath and phlegm–even though it might be anticipated that permanent workers, given their longer working hours, would experience greater health impacts. One plausible explanation is that temporary workers often face significant job insecurity, which has been linked to increased psychosocial stress and adverse health outcomes, including respiratory problems (36). Moreover, temporary employees are less likely to benefit from robust workplace safety measures and may be disproportionately allocated to more hazardous tasks or environments with higher levels of respiratory irritants (37, 38). These factors suggest that the cumulative effect of increased stress and poorer working conditions may predispose temporary workers to a higher risk of respiratory ailments.

The evidence presented in the research unequivocally establishes a direct link between high levels of dust exposure in the textile industry and adverse respiratory health outcomes among workers. Specifically, the association between respirable dust exposure and reduced lung function, alongside the elevated prevalence of respiratory conditions such as byssinosis, asthma, and chronic bronchitis, underscores the significant occupational health risks inherent in this sector (15, 39). Notably, even when particulate matter concentrations like PM10 and PM2.5 are within occupational safety thresholds, their chemical composition can still pose substantial health hazards, suggesting that current regulatory standards might not fully account for the complex nature of dust toxicity and its impact on respiratory health (40).

Furthermore, the compounded effects of duration of employment, specific work departments, and personal habits such as smoking on the severity of respiratory disorders among textile workers highlight the multifaceted nature of dust exposure risks (41, 42). This complexity is further exemplified by the moderate correlation between inhalable dust and endotoxin levels, indicating that adherence to dust exposure limits alone may not suffice in mitigating health risks, especially given the significant percentage of endotoxin samples exceeding workplace exposure limits (43).

Although there is an increasing trend of female labor force participation in the textile industry, women predominantly occupy manual labor roles with reduced wages. This wage disparity often nudges them to accept overtime, invariably in unhealthier conditions, culminating in severe health issues. The length of their tenure in such environments, as evidenced by the data, further accentuates the risk of chronic conditions such as respiratory issues and hypertension.

The prevailing trend of increased female labor force participation in the textile industry underscores a critical paradox; while it signifies progress toward economic engagement and autonomy for women, it simultaneously entraps them in a cycle of manual labor roles characterized by reduced wages and poorer working conditions (3). This dynamic is particularly pronounced in developing economies, where the textile sector emerges as a significant employer of women, offering a pathway out of poverty albeit at the cost of enduring exploitative labor practices and gender-specific discrimination (3, 44). Historical and regional analyses reveal that, despite the critical role of women in this sector, often in regions like Tamil Nadu, India, their employment is predominantly in low-skilled positions with poor remuneration, highlighting the gendered nature of employment within this industry (3).

This entrenched wage disparity and the gendered segmentation of labor roles not only perpetuate economic inequalities but also compel women to accept overtime work in pursuit of a livable income, frequently under unhealthier conditions (3, 44). Such circumstances invariably escalate the risk of severe health issues among female textile workers, reflecting a dire need for systemic changes to address these injustices. The evidence points toward a complex interplay between gender, employment, and health within the textile sector, necessitating targeted interventions to dismantle the structural barriers that perpetuate these disparities and ensure the wellbeing and economic empowerment of women in this critical industry.

Moreover, the study also touches upon the influence of non-occupational factors on health. Aspects such as separate kitchens and washrooms, age, and marital status play roles in moderating the risk of illnesses, indicating that personal and household characteristics intertwine with occupational factors in determining overall health outcomes. The interaction between occupational hazards and personal risk factors, including demographic characteristics, plays a pivotal role in determining workers' health outcomes. The amalgamation of workplace-related risks with individual predispositions such as genetics, age, gender, chronic disease prevalence, obesity rates, smoking habits, alcohol consumption, and prescription drug use, culminates in a pronounced impact on overall workforce health (45). Furthermore, psychosocial aspects of employment, such as concerns over job security, challenges in managing work-life balance, and experiences of workplace bullying or threats, are strongly correlated with adverse health evaluations among employees (46).

This study has several limitations. First, reliance on self-reported data for health symptoms, dust exposure classification (“dusty" vs. “less dusty"), and working conditions may introduce recall bias, subjective interpretation, or social desirability bias, potentially affecting the accuracy of associations between occupational exposures and health outcomes. The cross-sectional design precludes causal inference, as temporal relationships between exposures (e.g., work hours, contracts) and health effects remain unclear. Additionally, the absence of clinical validation for respiratory outcomes and medical histories limits the ability to account for pre-existing or acute respiratory conditions that could confound symptom reporting. Furthermore, key confounders such as indoor air pollution (e.g., cooking fuel type) and secondhand smoke exposure–both domestically and occupationally–were not comprehensively measured due to data constraints, potentially omitting influential variables. These limitations highlight the need for longitudinal designs, objective exposure assessments, and clinical diagnostics in future research.

## 5 Conclusion

The findings of this study illuminate the extensive prevalence of precarious work conditions within the textile mills of Pakistan. A significant proportion of women are subjected to environments that pose heightened risks of occupational diseases. These conditions are further exacerbated by the precariousness of their employment contracts and the fact that a substantial percentage earn wages that do not meet the national minimum standards. It is also imperative to note the association between various factors, such as extended working hours, the specific type of mill, the duration of employment, and certain household features, are correlated with the increased risk of respiratory symptoms. The implications of these correlations are profound, particularly in the context of formulating policies related to the textile industry. Specifically, these results underscore the urgent need for stricter enforcement of employment standards concerning minimum wages and maximum permissible weekly working hours.

## Data Availability

The raw data supporting the conclusions of this article will be made available on request by the authors, without undue reservation.
